# Initial Treatment: Prostaglandin Analog or Selective Laser Trabeculoplasty

**DOI:** 10.5005/jp-journals-10008-1114

**Published:** 2012-10-16

**Authors:** Colin I Clement

**Affiliations:** Glaucoma Unit, Sydney Eye Hospital, NSW, Australia; Central Clinical School Faculty of Medicine, The University of Sydney, NSW, Australia

**Keywords:** Prostaglandin analogs, Selective laser trabeculo-plasty, Glaucoma therapy, Compliance.

## Abstract

Prostaglandin analogs (PGA) have been the initial treatment of choice in many patients with glaucoma. However, there is an increasing awareness that non adherence and disruption of the ocular surface may limit PGA utility and tolerability respectively in some patients. In an eye with an open iridocorneal angle, these issues can potentially be addressed with the use of laser trabeculoplasty (LT). This therapy can achieve long-term intraocular pressure reduction following 1 to 2 treatment sessions without the ongoing need to apply medication (and preservatives) to the ocular surface. Whether PGAs or LT should be used in a given individual will also be influenced by other important factors including efficacy, response rate, tolerability, complications, cost and accessibility. This review examines these issues in relation to the initiation of primary therapy.

**How to cite this article:** Clement CI. Initial Treatment: Prostaglandin Analog of Selective Laser Trabeculoplasty. J Current Glau Prac 2012;6(3):99-103.

## INTRODUCTION

Prostaglandin analogs (PGA) have been the centerpiece of initial glaucoma management for many years due to their efficacy, once daily dosing and favorable side effect profile relative to other medications. In contrast, laser trabeculoplasty (LT) has had a less defined role in glaucoma management since its introduction in the 1980s.^1^ Many have used LT as a stopgap measure to avoid or delay surgery in eyes that are failing medical therapy. This was reflected in the study design for the advanced glaucoma intervention study (AGIS),^2^ where patients with advanced glaucoma were randomized to argon LT (ALT) followed by sequential trabeculectomies or trabeculectomy-ALT-trabeculectomy. Since then, LT has also been utilized in individuals intolerant of topical medications or in whom medication use is not practical (e.g. severe arthritis, restricting dexterity, chronic nonadherence).

However, LT has evolved from its initial development. Selective LT (SLT) represents a change in terms of ease of treatment (less specific target area, no need for posttreatment anti-inflammatory medication) although any difference in outcome or repeatability remains controversial and is probably not significant.^3^ Along with this has been an increasing awareness that glaucoma medications are often not used as intended and may severely compromise the health of the ocular surface. For these reasons, trabeculoplasty may be a better option for initial treatment in some patients.

### Efficacy and Response Rates

Head-to-Head Comparison

The intraocular pressure (IOP) lowering effect of latanoprost has been prospectively assessed against 90°, 180° and 360° SLT in a population with high baseline IOP.^4^ The most effective SLT regimen, 360° treatment, resulted in >20% IOP reduction in 82% of eyes and >30% in 59% of eyes. This was less than latanoprost in which IOP reduction >20% occurred in 90% of eyes and >30% in 78% of eyes, although this did not reach statistical significance. A similar prospective study^5^ compared 360° SLT with PGAs with 6 follow-up visits over 12 months. Outcome was measured in terms of reaching target IOP (as per the collaborative initial glaucoma treatment study) and number of additional steps needed to achieve target IOP. From a mean baseline of 24.5 to 24.7 mm Hg, there was no significant difference in IOP reduction between the two strategies but fewer interventions were needed in the SLT compared to medical group (11 *vs* 27%).

The soon to be released SLT/MED study, a prospective randomized trial of SLT *vs* prostaglandins, is expected to show equal efficacy after 12 months.

Nonresponders

Response to therapy may be defined as reaching target or desired IOP. The target IOP will differ depending on clinical need but a reduction of 20% is usually a minimum. Most studies report this over the short term and ignore the effects on other aspects of IOP, such as peak IOP or circadian IOP fluctuation. Despite this, target IOP and measuring response rates does provide valuable information about treatment efficacy.

The above-mentioned study by Nagar et al^4^ suggests that the nonresponder rate (defined as an IOP reduction of less than 20% from baseline) is approximately double for SLT compared to latanoprost (18 *vs* 10%). However, McIlraith et al^6^ did not report such a difference in a prospective comparison of SLT and latanoprost. Instead, they found similar response rates in both groups after 12 months (>20% reduction: 83% SLT, 84% latanoprost).

Ones overall impression is that the initial PGA non response rate is higher in these two studies than has been reported elsewhere. However, it is important to consider what happens over the longer term, when infact most studies report only short-term data (up to 3 months).^7^ There are in fact few studies that have examined PGA efficacy beyond 6 months. Of the few that extend for 6 months or more, there is suggestion of reducing response rates over time. A < 20% reduction in IOP was reported in approximately 13% of 128 eyes after 6 months of once daily latanoprost treatment in a multicenter randomized trial.^8^ With follow-up after 12 months of PGA treatment, nonresponders account for as much as 15 to 25% and it continues to decline from there.^9^ It is not clear whether these outcomes are subject to the same influence of nonadherence as in real-life; potentially these results could be worse.

Duration of Treatment Effect

The above-mentioned studies indicate that the PGA effect on IOP may wear off over time in some eyes. The 36-month study by Friström and Uusitalo^9^ suggests that treatment failure on PGAs is up to 40% by the end of this period. However, their definition of treatment failure was based on change to treatment that may occur even when IOP is low relative to baseline. Therefore, this likely over-represents treatment failure compared to a definition of IOP < 20% from baseline.

The long-term benefit of SLT as primary therapy for primary open angle glaucoma (POAG) and pseudoexfolia-tion glaucoma (PXFG) has been reported in a small prospective study.^10^ Treatment failure was defined as a return to baseline IOP (within 3 mm Hg) or initiation of further IOP-lowering treatment. Baseline IOP was 23.2 mm Hg (n = 19) for eyes with POAG and 25.5 mm Hg (n = 18) for eyes with PXFG. Mean follow-up was 27.1 months (range 6-42 months) for POAG and 20.4 months (range 3-42 months) for PXFG with considerable loss to follow-up in both groups for analysis beyond 30 months (42% POAG; 56% PXFG). By the 30 to 42 months analysis, mean IOP reduction was 5.7 mm Hg (24.6%) for POAG and 5.5 mm Hg (21.6%) for PXFG. By 30 months, failures accounted for 16% of POAG and 22% of PXFG.

Predictors of Outcome

Several studies point to baseline IOP as a predictor of IOP reduction after SLT.^11,12^ It is therefore not surprising that the efficacy of SLT in OAG with statistically normal IOP is not as good. El Mallah et al^13^ retrospectively analyzed response to SLT in 31 eyes of 18 patients with IOP < 22 mm Hg. Mean IOP reduction was 14.7% from a baseline IOP of 14.3 mm Hg. The number of eyes achieving a 20 or 30% IOP reduction was not reported and cannot be deduced from the presenting data.

Similarly, PGAs are less effective, when baseline IOP is less than 21 mm Hg.^14^ Further, Tsunda et al^15^ have shown that baseline IOPs < 15 mm Hg are associated with a smaller IOP reduction than IOPs in the 16 to 21 mm Hg range. To date, there are no studies that specifically compare IOP reduction in normal tension glaucoma between SLT and medication.

Adherence to therapy is directly related to treatment response meaning factors that influence adherence will have a knock on effect. Multiple risk factors for nonadherence have been identified and include personality type (depression, hypochondiasis), ethnicity (Afro-Caribbean, Latino), socioeconomic status (income, education, literacy), age, number of eye diseases and disease understanding.^16,17^ For this reason, LT may offer a better primary option in these patients. This is particularly so given interventions, such as telephone reminders and tailored print materials do not improve adherence rates^18^ and may be impractical for a health service to provide given the burden of glaucoma in the community.

### Adherence

One obvious advantage of LT is its ability to overcome the issue of nonadherence. Nonadherence remains a significant issue with glaucoma medication and this is certainly true for PGA use. Adherence to PGAs, regardless of which type, is estimated to be approximately 30% after 12 months.^19^ Adherence to medical treatment may be overrepresented in clinical trials due to the nature of the intervention and monitoring and also the patient characteristics of individuals willing to participate in trials. This raises the possibility that IOP reduction and any benefit derived from this in terms of visual performance may not be as good as is reported in the literature.

### Circadian IOP Fluctuation

There is increasing interest in IOP fluctuation and the role this plays in glaucoma pathogenesis.^20^ As such, it is interesting and important to consider treatment effect in terms of IOP fluctuation in addition to mean IOP reduction, percentage IOP reduction and nonresponse rates.

A number of studies have tried to assess the influence SLT has on IOP fluctuation. Prasad et al^21^ have examined the change in intervisit IOP fluctuation after 180 or 360° SLT. Their findings suggest intervisit IOP fluctuation is less following 360° treatment (IOP change < 2 mm Hg; 86%) compared with 180° treatment (IOP change < 2 mm Hg; 52%) but as intervisit fluctuation was not measured before treatment, no comment may be made about the overall treatment effect. The effect of SLT on diurnal IOP curves has been prospectively assessed.^22^ Twenty-six eyes not receiving medical therapy underwent 360° SLT then were subjected to repeat diurnal IOP curves at 3 and 6 months following. Interestingly, not a single eye achieved a mean diurnal IOP reduction of >20% in the 6 months of follow-up. Sixteen of 26 eyes were commenced on supplementary medical therapy because IOP reduction was thought to be insufficient. The remaining 10 eyes displayed a modest nonsignificant change in mean IOP after SLT (19.3 ± 1.4 mm Hg *vs* 18.6 ± 2.0 mm Hg) but a significant reduction in diurnal IOP fluctuation (7.2 ± 2.3 mm Hg *vs* 5.0 ± 1.7 mm Hg, p = 0.004).

The effect of latanoprost on intervisit IOP fluctuation has been reported. Following 6 months treatment, the rate of high fluctuation (defined as IOP > 6 mm Hg) reduced significantly from 22 to 6%.^23^

A direct comparison between latanoprost and SLT suggests PGAs may dampen IOP fluctuation more effectively.^24^ Comparison of diurnal IOP curves before and 6 months after treatment showed both strategies impact IOP fluctuation. SLT reduced IOP fluctuation by 41% from a mean of 5.5 ± 2.7 mm Hg. By comparison, latanoprost reduced IOP fluctuation by 64% from a baseline of 5.7 ± 2.1 mm Hg; the difference between groups was significant (p = 0.0444). Successful reduction in IOP fluctuation (defined as at least a 50% reduction from baseline) was achieved more often following latanoprost therapy (83%) compared to SLT (50%).

### Side Effects and Complications

Side effects reported following initiation of topical prostaglandins are extensive but are rarely severe.^25^ Well known side effects, include conjunctival hyperemia, hypertrichosis, iris hyperpigmentation and increased periocular skin pigmentation. Others include pruritis, cataract, eyelid edema, foreign body sensation and eye pain. In addition, topical glaucoma medications, including prostaglandin analogs are associated with an increased rate of ocular surface disease (OSD).^26,27^

Complications after SLT are few. The commonest is probably mild anterior chamber inflammation that is transient and requires no treatment (48%, McIlraith et al^6^ 83%, Latina et al^28^). IOP spikes of 5 or 8 mm Hg have been reported in 25 and 9% of treated eyes respectively (Latina et al^28^), however, none of these persisted beyond 24 hours. Comparable rates of IOP elevation after SLT have been reported by Nagar et al^4^ (>5 mm Hg, 27%), however, lower rates are reported in other studies by Damji et al^29^ (>6 mm Hg, 4.5%) and Lai et al (>5 mm Hg, 10.3%). Ocular discomfort is sometimes reported following SLT (15%, Latina et al;^28^ 39%, Nagar et al^4^) but this effect does not last.

### Cost and Availability

The cost of treating with medications or SLT has recently been reported.^30^ This analysis using data from the USA looked at cost from a patient perspective assuming that both eyes received treatment, SLT was applied to 360° in a single session and SLT was associated with posttreatment uveitis and IOP spikes in 50 and 27% respectively. They found the cost of SLT equalled branded PGAs after 6.3 to 6.8 months of treatment and generic latanoprost by 13.1 months. Therefore, it was concluded that if SLT is applied every 6 to 12 months, it maintains cost equivalence with PGA eyes drops. As IOP reduction is maintained beyond 12 months in many patients without the need for further intervention, SLT may actually be a more affordable option.

This analysis did not model for the costs of additional glaucoma therapy, including other medical treatment, complications after SLT or the need for glaucoma surgery. It also did not take into account the cost of patient transport and repeat visits nor the effect medical treatment has on OSD. Both medical treatment and SLT costs may be affected by these variables. However, this analysis does highlight the potential cost savings of SLT treatment in individuals that display an extended response to treatment.

An attempt has been made to take these extra factors into account. Using Markov mathematical modeling, Stein et al^31^ have attempted to estimate the cost comparison over 25 years of starting a patient with mild glaucoma on medical therapy verses LT. The model takes into account the progressive and incremental nature of glaucoma and also the cost of assessment, annual supply of medications, laser or incisional surgery, accessing low-vision services as well as complications and adverse events. It was concluded, when nonadherence is taken into account, that LT is more economical.

### Patient Limitations

SLT cannot be performed in closed or very narrow angles whereas this does not prevent the use of medications. There may be other physical limitations that prevent the use of SLT, including severe kyphosis, ankylosing spondylosis, torticollis or cervical arthritis preventing head placement on the laser unit. Head tremor prevents accurate placement of the laser treatment and eyes that are deeply recessed or have narrow palpebral apertures sometimes make gonioscopy lens placement difficult. This is also true in patients with moderate to severe blepharospasm.

## SUMMARY

Data on the comparative efficacy of SLT and PGA therapy for initial IOP lowering is limited although the forth coming SLT/MED study will in part address this knowledge gap. Currently it appears both treatments have similar efficacy but further studies are needed to definitively answer this question. In the intermediate to long term, SLT may have the edge over PGAs in terms of cost of treatment and is an important consideration as the burden of glaucoma increases. SLT has a number of advantages in the initial management of open-angle glaucoma or ocular hypertension, including its ability to overcome the significant issue of treatment nonadherence that is seen with topical medical treatments. SLT reduces the burden of daily medical treatment; this may be more detrimental to quality of life than the condition itself. However, in a condition that is often asymptomatic, medications may have an important role to play in terms of reminding the patient about their condition and the need for vigilance. There is a risk that patients may perceive SLT to be a definitive treatment resulting in complacency with regard to ongoing assessment. Further, PGAs may have greater benefits in terms of their effect on circadian IOP changes. However, our understanding of how this influences glaucoma progression is still evolving so the significance of this effect is not yet known. There are patient groups that are more suited to either treatment and the ultimate decision requires the input of both physician and patient. Patient factors, past experience and local resources should all be considered, when offering initial therapy.
